# Supplementary material for: Multi‐dataset identification of innovative feature genes and molecular mechanisms in keratoconus
Source: J Cell Mol Med. 2024 Sep 19;28(18):e70079. doi: 10.1111/jcmm.70079 (PMC11412914; doi:10.1111/jcmm.70079)
Supplement: Supplementary file 2 — Material S2. [file JCMM-28-e70079-s002.zip › 7.GSEA analysis/4.MYRF/KEGG_TYROSINE_METABOLISM.html]

Details for gene set KEGG\_TYROSINE\_METABOLISM[GSEA]

|  || Dataset | MYRF.group.cls#h\_versus\_l |
| Phenotype | group.cls#h\_versus\_l |
| Upregulated in class | l |
| GeneSet | KEGG\_TYROSINE\_METABOLISM |
| Enrichment Score (ES) | -0.5138073 |
| Normalized Enrichment Score (NES) | -1.675993 |
| Nominal p-value | 0.004040404 |
| FDR q-value | 0.20612453 |
| FWER p-Value | 0.447 |
Table: GSEA Results Summary

  

Fig 1: Enrichment plot: KEGG\_TYROSINE\_METABOLISM      
 Profile of the Running ES Score & Positions of GeneSet Members on the Rank Ordered List

  

| SYMBOL | TITLE | RANK IN GENE LIST | RANK METRIC SCORE | RUNNING ES | CORE ENRICHMENT || 1 | AOC2 | na | 1119 | 0.443 | 0.0149 | No |
| 2 | ADH6 | na | 1268 | 0.424 | 0.0557 | No |
| 3 | TAT | na | 1333 | 0.416 | 0.0982 | No |
| 4 | IL4I1 | na | 4670 | 0.233 | 0.0268 | No |
| 5 | TPO | na | 4921 | 0.225 | 0.0435 | No |
| 6 | ADH1B | na | 5054 | 0.221 | 0.0631 | No |
| 7 | ADH4 | na | 5377 | 0.211 | 0.0763 | No |
| 8 | PNMT | na | 7021 | 0.163 | 0.0463 | No |
| 9 | ALDH3B1 | na | 7089 | 0.161 | 0.0616 | No |
| 10 | MAOB | na | 8655 | 0.124 | 0.0296 | No |
| 11 | METTL2B | na | 10616 | 0.085 | -0.0179 | No |
| 12 | TRMT11 | na | 10871 | 0.080 | -0.0167 | No |
| 13 | GOT2 | na | 11639 | 0.069 | -0.0315 | No |
| 14 | GSTZ1 | na | 12168 | 0.061 | -0.0402 | No |
| 15 | LCMT2 | na | 13654 | 0.042 | -0.0786 | No |
| 16 | ADH7 | na | 14404 | 0.033 | -0.0966 | No |
| 17 | TH | na | 14693 | 0.031 | -0.1016 | No |
| 18 | COMT | na | 15993 | 0.018 | -0.1372 | No |
| 19 | GOT1 | na | 16062 | 0.017 | -0.1373 | No |
| 20 | DDC | na | 16630 | 0.012 | -0.1524 | No |
| 21 | MAOA | na | 16972 | 0.009 | -0.1613 | No |
| 22 | FAH | na | 17956 | -0.000 | -0.1896 | No |
| 23 | LCMT1 | na | 18916 | -0.009 | -0.2163 | No |
| 24 | MIF | na | 19281 | -0.014 | -0.2253 | No |
| 25 | ALDH3A1 | na | 24123 | -0.078 | -0.3566 | No |
| 26 | ALDH3B2 | na | 25910 | -0.111 | -0.3963 | No |
| 27 | METTL6 | na | 26260 | -0.119 | -0.3937 | No |
| 28 | ADH1A | na | 26960 | -0.134 | -0.3996 | No |
| 29 | ADH5 | na | 27321 | -0.143 | -0.3948 | No |
| 30 | HGD | na | 31401 | -0.286 | -0.4820 | Yes |
| 31 | DCT | na | 32507 | -0.351 | -0.4765 | Yes |
| 32 | ALDH1A3 | na | 32684 | -0.364 | -0.4428 | Yes |
| 33 | HEMK1 | na | 32824 | -0.374 | -0.4070 | Yes |
| 34 | AOX1 | na | 33262 | -0.415 | -0.3755 | Yes |
| 35 | ADH1C | na | 33352 | -0.425 | -0.3328 | Yes |
| 36 | DBH | na | 33453 | -0.437 | -0.2892 | Yes |
| 37 | AOC3 | na | 34487 | -0.672 | -0.2475 | Yes |
| 38 | TYRP1 | na | 34607 | -0.755 | -0.1705 | Yes |
| 39 | TYR | na | 34631 | -0.783 | -0.0879 | Yes |
| 40 | HPD | na | 34668 | -0.852 | 0.0017 | Yes |
Table: GSEA details [plain text format]

  

Fig 2: KEGG\_TYROSINE\_METABOLISM      
 Blue-Pink O' Gram in the Space of the Analyzed GeneSet

  

Fig 3: KEGG\_TYROSINE\_METABOLISM: Random ES distribution      
 Gene set null distribution of ES for **KEGG\_TYROSINE\_METABOLISM**
